# Rescue of autoimmune hepatitis by soluble MHC class II molecules in an altered concanavalin A‐induced experimental model

**DOI:** 10.1002/ame2.12133

**Published:** 2020-08-27

**Authors:** Katerina Bakela, Maria Georgia Dimitraki, Evangelia Skoufa, Irene Athanassakis

**Affiliations:** ^1^ Laboratory of Immunology Department of Biology University of Crete Heraklion Crete Greece

**Keywords:** autoimmune hepatitis, cytokines, immunosuppression, inflammation

## Abstract

**Background:**

Soluble major histocompatibility complex class II (sMHCII) molecules have been described to maintain tolerance through the suppression of autoreactive T lymphocytes. In order to evaluate their ability to rescue autoimmune hepatitis (AIH) symptoms, the present work attempted to administer sMHCII molecules to an in vitro as well as in vivo concanavalin A (ConA)‐induced AIH model.

**Methods:**

The in vitro AIH model consisted of splenocyte stimulation with ConA in the presence or absence of serum‐isolated sMHCII molecules. An in vivo ConA‐modified model with or without sMHCII treatment was developed. The cytokine profile in culture supernatants and serum was tested by ELISA. Cell markers were evaluated by immunofluorescence, while cell proliferation by tritiated thymidine uptake. AIH symptoms were assessed by daily observations for the establishment of a disease severity scoring system and liver histology was evaluated using a biomolecular imager.

**Results:**

The presence of sMHCII molecules in the ConA‐stimulated cell cultures leads to a significant reduction of cell proliferation. The administration of sMHCII molecules to the ConA‐treated animals showed a significant reduction in the levels of IL‐2, IL‐4, and IL‐10, as well as a decrease in the number of spleen CD4^+^ and CD8^+^ cells. Upon development of a scoring system, it was shown that the sMHCII treatment was accompanied by a slower progression of the disease, while rescuing fibrotic liver morphology.

**Conclusion:**

The results presented in this study confirm the ability of sMHCII proteins to alleviate autoimmune hepatitis, possibly highlighting new therapeutic approaches for autoimmune diseases.

## Graphical abstract

Rescue of autoimmune hepatitis symptoms by serum soluble major histocompatibility complex class II (sMHCII) molecules in a concanavalin‐A‐induced murine experimental model.

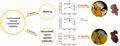

## 1 INTRODUCTION

Autoimmune hepatitis (AIH) is a chronic inflammatory disease of the liver that occurs globally (2:100.000 per year) in all ethnicities, affecting children and adults of all ages, with a female predominance.^1^ It is characterized by a loss of self‐tolerance leading to the appearance of autoantibodies, histological changes, and pathological dysfunctions. The diagnosis of AIH requires a combination of biochemical and histological tests, usually relying on the presence of autoantibodies (antinuclear antibodies‐ANAs, anti‐smooth muscle antibodies‐SMAs, antibodies to the soluble liver antigen‐LP, liver/kidney microsomal antibodies‐LKKM‐1, peripheral antinuclear neutrophil antibodies, liver cytosol type 1 antibodies, liver‐specific membrane lipoprotein antibodies, etc) in the serum, increased serum transaminase levels, increased numbers of immune cells or characteristic formations on liver histology, which also distinguish type 1 from type 2 AIH.^2^, ^3^, ^4^, ^5^ The precise etiology of AIH is yet to be elucidated, but an interaction between genetic and environmental factors is regarded as the main underlying pathogenic mechanism. AIH seems to arise in genetically susceptible individuals when triggered by exposure to pathogens, xenobiotics, or other stimuli, leading to T‐cell‐mediated autoimmune response directed against liver autoantigens, most possibly due to molecular mimicry, which, due to inadequate regulatory immune control, leads to loss of tolerance.^3^, ^6^, ^7^ The dominant cells to be activated appear to be the CD4^+^ T‐cell populations, and to a lesser degree, CD8^+^ and NK cells.

A variety of induced and genetically manipulated experimental models has been developed to study liver immunology and autoimmune‐mediated liver damage, so that novel therapeutic strategies could be developed for the human disease. The most commonly induced models apply endotoxins and plant lectins including lipopolysaccharide in the presence or not of D‐galactosamine and concanavalin A (ConA),^8^, ^9^ while genetically manipulated models include usually the overexpression of specific proteins under systemic or organ specific expression or knocking‐out of one or more proteins.^10^, ^11^, ^12^, ^13^, ^14^ The present work concentrated on the ConA‐induced experimental model, which has been thoroughly studied vis‐à‐vis the immune mechanisms governing autoimmune hepatitis.^9^ Compared to the other available models,^10^, ^11^, ^12^ the ConA‐induced AHI model has certain benefits: (a) ConA is an economic inducer, (b) acts as mitogen for T lymphocytes (ideal for in vitro trials), (c) induces a rapid and easy development of autoimmunity that is mediated by significant changes in cytokine levels, offering thus an easy way to confirm the development of the disease, (d) offers absolute and repeatable results minimizing thus the need of large numbers of animals, and most importantly, (e) the ConA‐induced AIH model appears to mimic the actual human AIH condition the best, as it involves activated T lymphocytes, resembling the autoreactive T cells in the human disease.

The available therapeutic approaches for AIH, similarly to any other autoimmune condition, are characterized as inadequate and not personalized. The widespread therapeutic approach to date consists mainly of immunosuppressive drugs, corticosteroids (prednisone or prednisolone) at high initial doses, followed by azathioprine.^15^, ^16^, ^17^ Although, corticosteroids are substances with very generalized action and a number of side effects, hence, their prolonged use can, in long term, lead to weight gain, cosmetic changes (including acne), brittle diabetes, cataract, psychosis, osteoporosis, high blood pressure, increased risk of infection, liver damage, decreased fertility, and increased risk of cancer.^16^ Thus, systemic immunosuppression cannot be considered as a treatment for the disease, since despite the alleviation of autoimmune symptoms, most of the times fails to re‐establish tolerance. Although it is considered that prolonged immunosuppression can deplete autoreactive T‐cell clones, side effects do not allow to follow such therapies for long periods of time. In addition, apart from the side effects, immunosuppressants are not effective for all patients, due to a very high clinical heterogeneity that makes it almost impossible to find a suitable, universal therapeutic protocol.^18^ In extreme cases, where the patient's progress to end‐stage liver disease, liver transplantation is a life‐saving option, although AIH can recur or develop de novo after transplantation.^19^, ^20^ Therefore, there is an imperative need for a novel treatment that combines the absence of side effects with high efficiency.

Recent observations indicate that soluble major histocompatibility complex class II (sMHCII) molecules found in serum and body fluids of healthy individuals, among their other properties, play an important role in maintaining tolerance.^21^ In particular, sMHCII molecules are proteins of approximately 60 kD that appear to compete with membrane MHCII for T‐cell receptor (TCR) binding, leading to preclusion of T‐cell activation and interception of inflammatory cytokine production, thus, driving the immune system toward suppression. Self‐tolerance is believed to be mediated by sMHCII molecules loaded with self‐antigens, which bind to the TCR of autoreactive T lymphocytes leading to suppression and the overall protection from autoimmunity.^22^

The suppressive properties of sMHCII molecules have previously been tested in an experimental murine model of systemic lupus erythematosus (SLE), where the administration of sMHCII molecules leads to a gradual decrease of autoantibodies, activation of suppressive T‐cell populations and alleviation of the disease symptoms.^23^

Based on the above observations the goal of the present study was to apply the sMHCII immunosuppressive treatment to an AIH mouse experimental model and examine whether such manipulation could have a beneficial effect on the development of the disease in vitro as well as in vivo. To this extend, a modified ConA model was developed to achieve long‐lasting and milder symptoms of the disease, which could provide the necessary timing for treatment. The results presented herein show that indeed sMHCII proteins could reverse the serologic and cellular markers to control levels and rescue liver damage in the ConA‐induced AIH model.

## 2 MATERIALS AND METHODS

### 2.1 Animal experimentation

BALB/c (H‐2^d^) inbred mice were purchased from Charles River (Milan, Italy) and bred in the animal facility of the Department of Biology at the University of Crete (Crete, Greece, EL91‐BIObr‐09) under standard conditions of temperature (18‐25°C), humidity (45%‐50%), and photoperiod of 12 hours light and 12 hours dark. Males 6‐8 weeks of age were handled according to the international and national bioethical rules and conformed to the bioethics regulations following the EU Directive 2010/63/EU for animal experiments. In the context of the 3Rs principles of animal welfare, 4‐10 mice were assigned, depending on the experimental repetitiveness. Mice were sacrificed by cervical dislocation and spleens were removed under aseptic conditions.

For the in vivo induction of AIH, BALB/c mice received an intraperitoneal injection/wk of 20 mg/kg ConA for 2 weeks. Mice were submitted to tail bleeding every week prior and 3 hours after ConA injection. The production of cytokines IL‐2, IL‐4, IFN‐γ, and IL‐10 in the serum was examined in order to evaluate disease development. Serum was used at the dilution of 1/1000 in ELISA experiments.

### 2.2 Antibodies

Mouse anti‐IA/IE mAb (HB‐225™ hybridoma: *Mus musculus* (myeloma), hamster, Armenian B cell, reacts with a monomorphic determinant on the I‐A and I‐E region, IgG isotype, generous gift from Dr R Steinman, Rockefeller University, NY) was purified from culture supernatants, used at the concentration 0.1 μg/mL in ELISA experiments and was covalently linked to magnetic beads coupled with Sheep anti‐Mouse IgG for protein purification procedures (see below).

For immunofluorescence experiments, FITC‐labeled rat anti‐mouse CD4 (IgG2b, Eurobioscience, Germany) and FITC‐labeled rat anti‐mouse CD8 (IgG2α, k, Biolegend, San Diego, CA) were used at the concentration of 1 µg/mL. Furthermore, rat anti‐mouse IL2 (IgG2a, k, Biolegend), rat anti‐mouse IL4, IL‐6, IL‐10, and IFN‐γ (IgG1, k, Biolegend), were used at the concentration of 0.1μg/mL in ELISA experiments.

### 2.3 Purification of sMHCII proteins

Dynabeads M‐280 Sheep anti‐mouse IgG (Dynabeads M‐280, 2.8 μm superparamagnetic beads with affinity purified polyclonal sheep anti‐mouse IgG1, IgG2a, IgG2b, Life Technologies, Carlsbad, CA) were cross‐linked with the mouse anti‐IA/IE HB‐225™ mAb and were used for the isolation of sMHCII proteins following the instructions of the manufacturer as previously described.^22^ Briefly, 10^8^ Dynabeads M‐280 Sheep anti‐Mouse IgG were coupled to 5 μg HB‐225™ with rotational mixing for 60 minutes at 4°C. After washing the beads twice using a magnet with 1‐mL PBS (pH 7.2), 1 mL of 0.2 mol/L triethanolamine (pH 8.2) was added to the magnetic beads with the immobilized HB‐225™. The beads were thereafter washed twice with 1 mL of 0.2 mol/L triethanolamine (pH 8.2) resuspended in 1 mL of 20 mmol/L dimethyl pimelimidate dihydrochloride (DMP, Pierce, Rockford, IL, USA) in 0.2 mol/L triethanolamine, pH 8.2 (5.4 mg DMP/mL buffer) and incubated with rotational mixing for 30 minutes at 25°C. After removing the supernatants, the reaction was stopped by resuspending the beads in 1 mL of 50 mmol/L Tris, pH 7.5 and incubating for 15 minutes with rotational mixing. The cross‐linked Dynabeads were washed three times with 1‐mL PBS, resuspended in 1‐mL mouse serum (1:1 v/v in PBS) and incubated with rotational mixing for 2 hours at 4°C. After washing twice with 1‐mL PBS, elution was performed using 2‐mol/L NaCl, with rotational mixing for 20 minutes at 25°C. The recovered sMHCII protein was dialyzed against PBS and concentrated using centrifuge filters (cut off 10 000 Da; centricon 10; Amicon Inc, Beverly, MA).

### 2.4 SDS‐PAGE gel

Purified (as described in the Section 2.3) sMHCII protein (20 μL) was loaded onto a 12% SDS‐polyacrylamide gel and run in an electrophoresis apparatus (GibcoBRL). Proteins were visualized using silver stain,^24^ which is the most sensitive colorimetric method for detecting total protein, ensuring thus purity of the isolated preparation.

### 2.5 ELISA techniques

Indirect ELISA was performed in order to verify sMHCII molecule purification and detect IL2, IL‐4, and IL10 cytokines in mouse serum, as previously described.^22^

### 2.6 Cell proliferation assays

Spleen cells were cultured in 96‐well V‐bottomed plates (Sarstedt, Numbrecht, Germany) at the concentration of 1 × 10^6^ cells/well in DMEM culture medium (Biosera, Kansas City, USA) supplemented with 10% fetal bovine serum (FBS, Gibco) at a final volume of 200 μL with or without ConA^7^, ^9^ (1 μg/mL) and with or without sMHCII (30 ng/mL)^25^ and processed for ^3^HTdR incorporation assays after 4 days of culture as previously described.^22^

### 2.7 Immunofluorescence of spleen cells

Spleen cells were collected and upon elimination of red blood cells, they were washed twice with PBS 1 mL and incubated with PBS‐Bovine Serum Albumin (BSA) 3% buffer for blocking the remaining protein‐free sites at room temperature for 30 minutes. After washing with PBS, the cells were incubated with the conjugated antibodies diluted in PBS‐BSA 1% at room temperature for 45 minutes. After washing, the cells were resuspended in PBS and processed to flow cytometry analysis.

### 2.8 Tissue isolation and scanning

Liver tissues were isolated 6 months after the ConA and the ConA + sMHCII treatment of mice and fixed using PFA 4%, under rotation, at 4°C for 24 hours. Azure Biosystems Sapphire™ Biomolecular Imager (Azure Biosystems, Dublin, CA 94568 USA) was used in order to scan the liver tissues in 10‐µm resolution. This instrument combines NIR fluorescence (both long and short), RGB fluorescence, chemiluminescent, and phosphor imaging capabilities, while using four solid state lasers as excitation sources (450, 520, 660, and 780 nm). The application of the four‐channel fluorescence mode at 10‐μm resolution, could detect gross anatomy and morphology of liver tissues, mainly based on tissue autofluorescence.

### 2.9 Statistical analysis

Data were analyzed using two‐tailed Paired (in vitro experiments) or Unpaired *(*in vivo experiments) Student's *T* test. *P*‐values < .05 were considered significant (*), values <.01 were considered very significant (**), and values <.001 and <.0001 were considered highly significant (*** and ****). Statistics were performed using GraphPad Prism 7 (Graphpad Software, La Jolla, CA).

## 3 RESULTS

In an effort to alleviate autoimmune hepatitis symptoms using sMHCII molecules, in vitro as well as in vivo murine experimental autoimmune hepatitis models were designed.

### 3.1 Development of an in vitro autoimmune hepatitis experimental model

Concentrating on the antigenic stimulus of ConA in the development of AIH model, the effect of sMHCII molecules was initially examined on ConA‐activated spleen cells.

**FIGURE 1:**
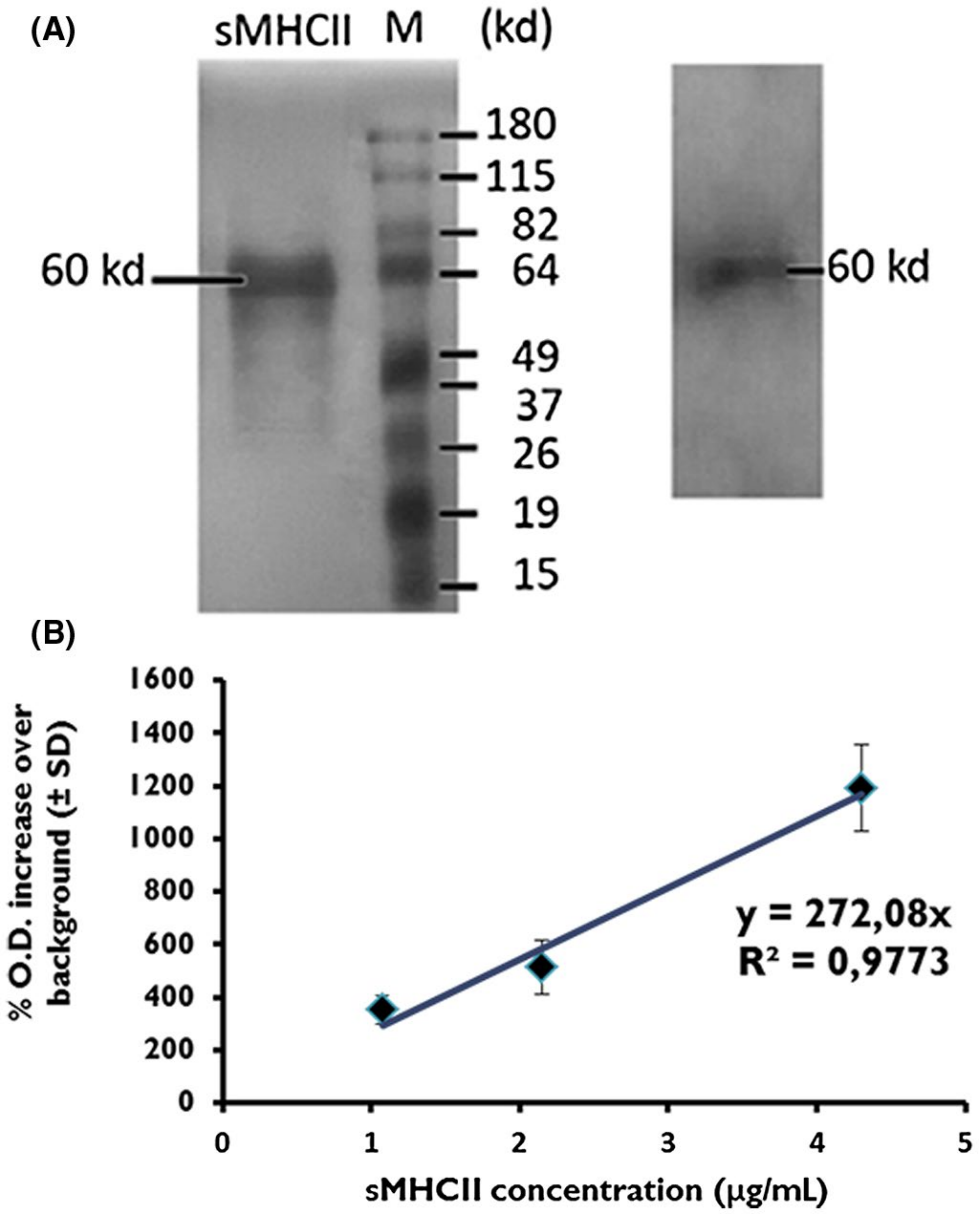
Isolation and purification of sMHCII proteins. Soluble MHCII proteins were isolated from serum of healthy BALB/c mice using a magnetic bead isolation procedure (see Section 2.3). Upon purification, sMHCII proteins were submitted to SDS‐PAGE electrophoresis (A) and ELISA experiments (B). One representative experiment out of 10 is shown

To this extend, sMHCII proteins were purified from the serum of healthy BALB/c mice, using magnetic bead protein isolation protocols. As previously described,^22^ single band proteins could be detected in silver stained SDS‐page gel electrophoresis experiments at a molecular weight of 60 kD (Figure 1A). The isolated proteins were concentrated, dialyzed against PBS and tested for their specificity by ELISA (Figure 1B). Protein concentration was evaluated using the Lοwry protein determination assay. These preparations were used thereafter for all experiments.

**FIGURE 2:**
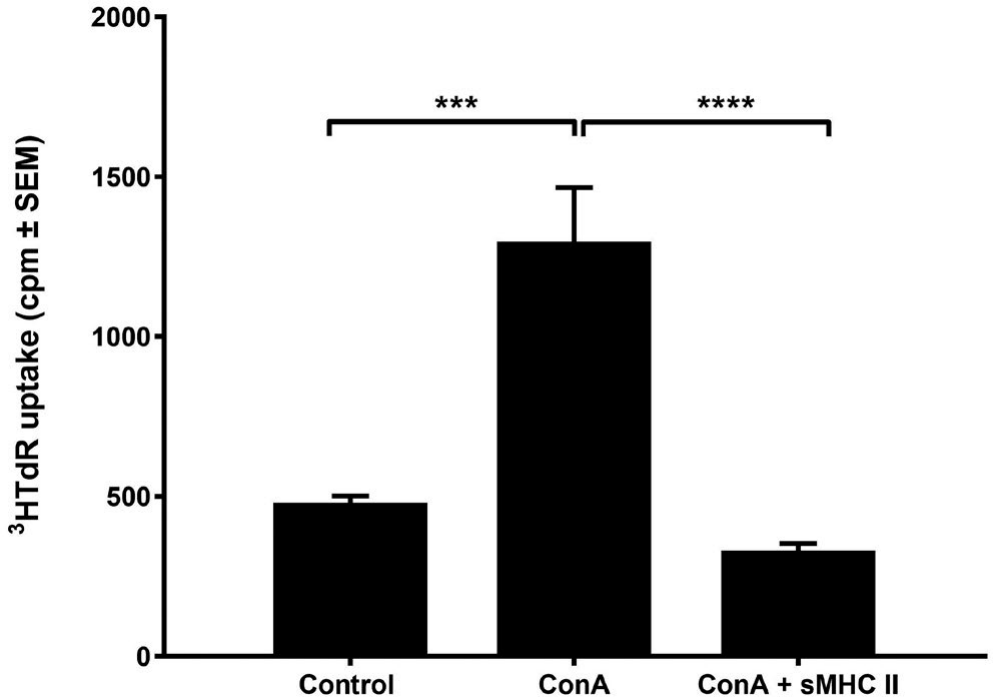
Effect of ConA in the presence or not of sMHCII on spleen cell proliferation. Spleen cells from BALB/c mice were incubated with 1 μg/mL ConA in the presence or not of 30 ng/mL sMHCII and examined as to their proliferative activity using ^3^HTdR uptake techniques. The results represent the mean of six different experiments and are expressed as cpm ± SEM. ***P < .0003, ****P < .0001

The mitogenic activity of ConA as to its ability to stimulate T cells is well documented.^26^ Thus, naïve spleen cells from BALB/c mice were incubated with the suboptimal dose of 1 µg/mL ConA and 1 day after culture initiation sMHCII were added at a concentration of 30 pg/mL, which has been previously shown to provide best proliferative activity.^25^ Upon culture termination (day 4; considered as the best timing for testing profileration^26^), the cells were tested for their proliferative activity using ^3^HTdR uptake techniques. The results showed a statistically significant 172.6% increase (*P* < .0003) of cell proliferation in response to ConA as compared to controls, whereas the presence of sMHCII significantly decreased by 207% ^3^HTdR uptake (*P* = .0001) as compared to the ConA‐treated controls (Figure 2).

Since sMHCII could suppress the conA‐induced spleen cell proliferation in vitro, it was then inquired whether similar effects could be observed in vivo.

### 3.2 Development of an in vivo ΑΙΗ experimental model

**FIGURE 3:**
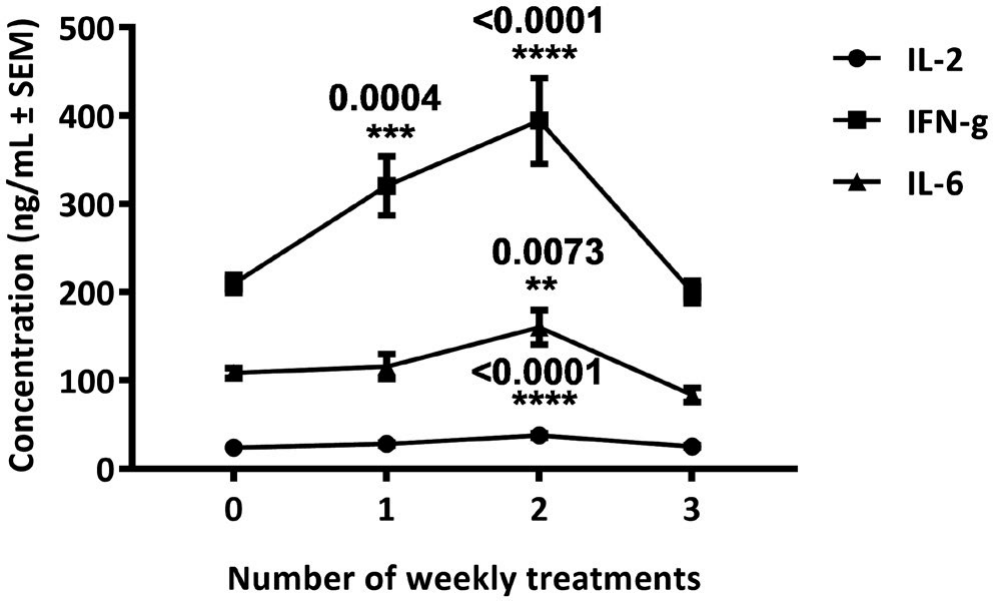
Establishment of an in vivo AIH model. BALB/c mice received 1, 2, or 3 intraperitoneal injections of 20 mg/kg ConA within weekly intervals. Three hours after each injection, blood was collected. The presence of IL‐2, IFN‐γ, and IL‐6 in the serum was detected by ELISA. The results represent the mean of 10 animals tested and are expressed as ng/mL ± SEM. Statistical significance is shown by asterisks and* p*‐values

The experimental AIH model implicated herein consisted of 20 mg/kg ConA administration to BALB/c mice.^10^, ^11^ The choice of the specific mouse strain was based on its susceptibility to the ConA treatment. Strains like C57BL/6 and C3H/He are considered to be highly susceptible and apparently time‐limiting for the application of a therapeutic protocol.^8^ In contrast to the literature‐described intravenous injection of ConA, in the present study intraperitoneal administration was performed. The so far described experimental model for AIH using intravenous ConA administration is a quite rapid and aggressive scheme of treatment, therefore animals were in most cases sacrificed 8 hours after injection.^8^ Such protocol could not be useful to the experimental design of the present study, since it does not leave enough time for testing rescuing of the pathologic phenotype by the proposed treatment. Therefore, in an effort to obtain a milder AIH phenotype, 1, 2, or 3 intraperitoneal injections of ConA were performed within weekly intervals, and the effectiveness of the treatment was tested by examining critical cytokine levels in the serum. As suggested by the literature, serum IL‐2, IFN‐γ, TNF‐α, IL‐4, IL‐10, IL‐12, and IL‐6 could define the development of AIH.^11^ Among these cytokines, IL‐2, IL‐4, and IL‐10 were showing higher levels 3 hours after ConA administration.^11^ Concentrating on the inflammatory cytokines IL‐2, IFN‐γ, and IL‐6, these were examined in the protocols of 1, 2, or 3 intraperitoneal ConA injections 3 hours after the last dose of treatment. The results showed that serum IL‐2, IFN‐γ, and IL‐6 in mice having received the 2‐week treatment showed a statistically significant increase of 58 (*P* < .001), 78.6 (*P* < .001), and 35.7% (*P* = .0073) as compared to controls (Figure 3). Among these cytokines, IL‐2 and IFN‐γ showed a statistically significant increase (17% *P* = .0004 and 48.3%, *P* = .004, respectively) on the first week of treatment (Figure 3). Therefore, based on these results, it was considered that the 2‐week treatment could be the best protocol for slower and amendable manifestation of the AIH phenotype.

### 3.3 Rescuing the AIH phenotype by sMHCII

**FIGURE 4:**
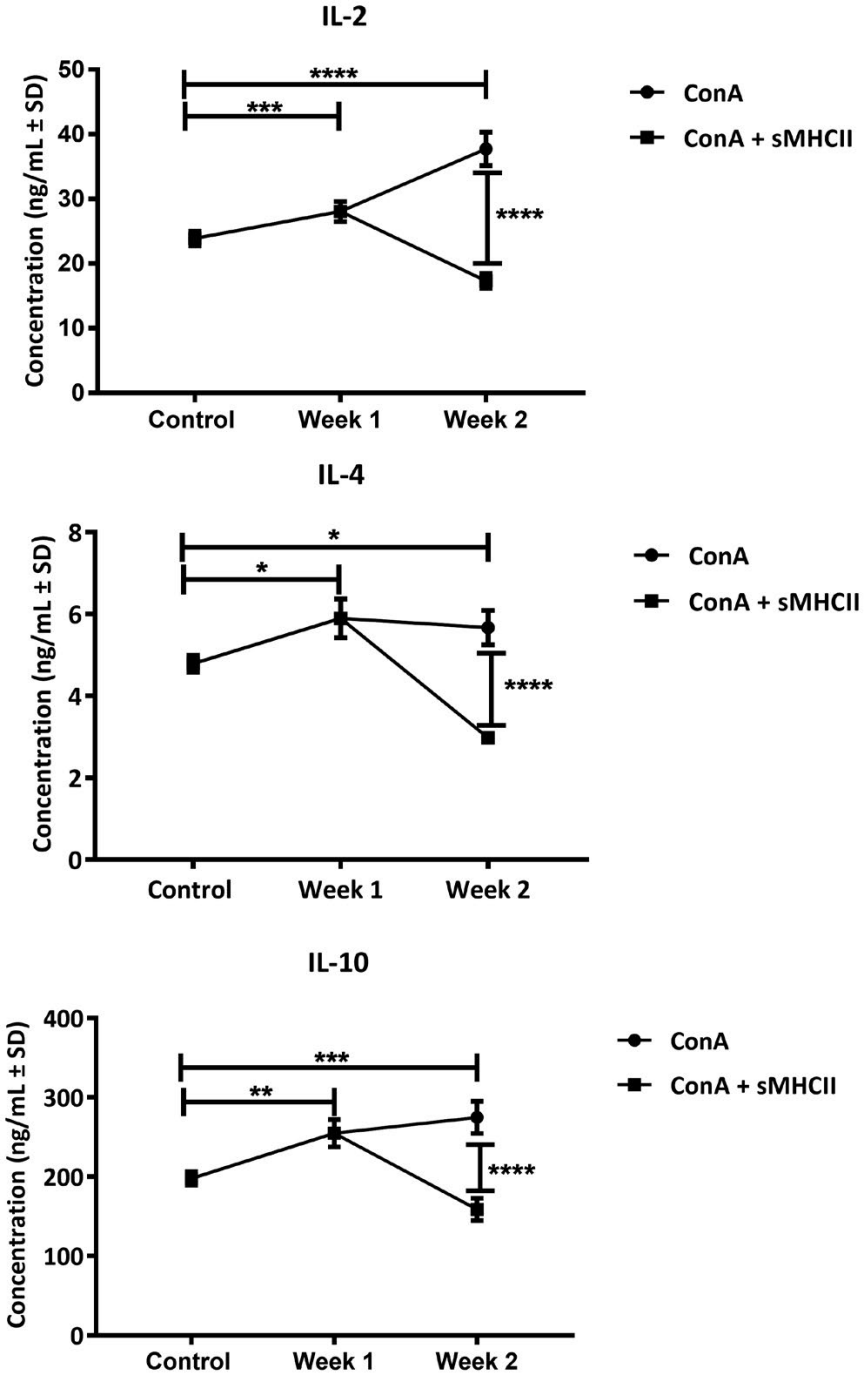
Effect of sMHCII administration on serum interleukin content of ConA‐treated animals. ConA‐treated animals received 0.3 μg/gr of body weight sMHCII and IL‐2, IL‐4, and IL‐10 in the serum was evaluated 3 h later by ELISA experiments. The results represent the mean of eight animals in each case and are expressed as ng/mL ± SEM. *P < .02, **P < .002, ***P < .0005, ****P < .0001

Concentrating on the 2‐week treatment protocol, it was then inquired whether sMHCII proteins could rescue cytokine increase in the serum. In this case, in order to include typical Th1 ad Th2 cytokines in the study, IL‐2, IL‐4, and IL‐10 were examined in the serum. The levels of the above cytokines showed a statistically significant increase after the first and second ConA injection within the week interval (*P* = .0004 and <.0001, *P* = .0295 and .0363, *P* = .0042 and .0001 on the first and second week of the ConA treatment for IL‐2, IL‐4 and IL‐10, respectively; Figure 4). Three hours after the second injection of ConA, mice received 0.3 μg/gr of body weight of sMHCII.^23^ In all cases, the presence of sMHCII significantly decreased (*P* < .0001) by 54, 47.5 and 42.2% for IL‐2, IL‐4, and IL‐10, respectively, as compared to the ConA‐treated animals (Figure 4).

**FIGURE 5:**
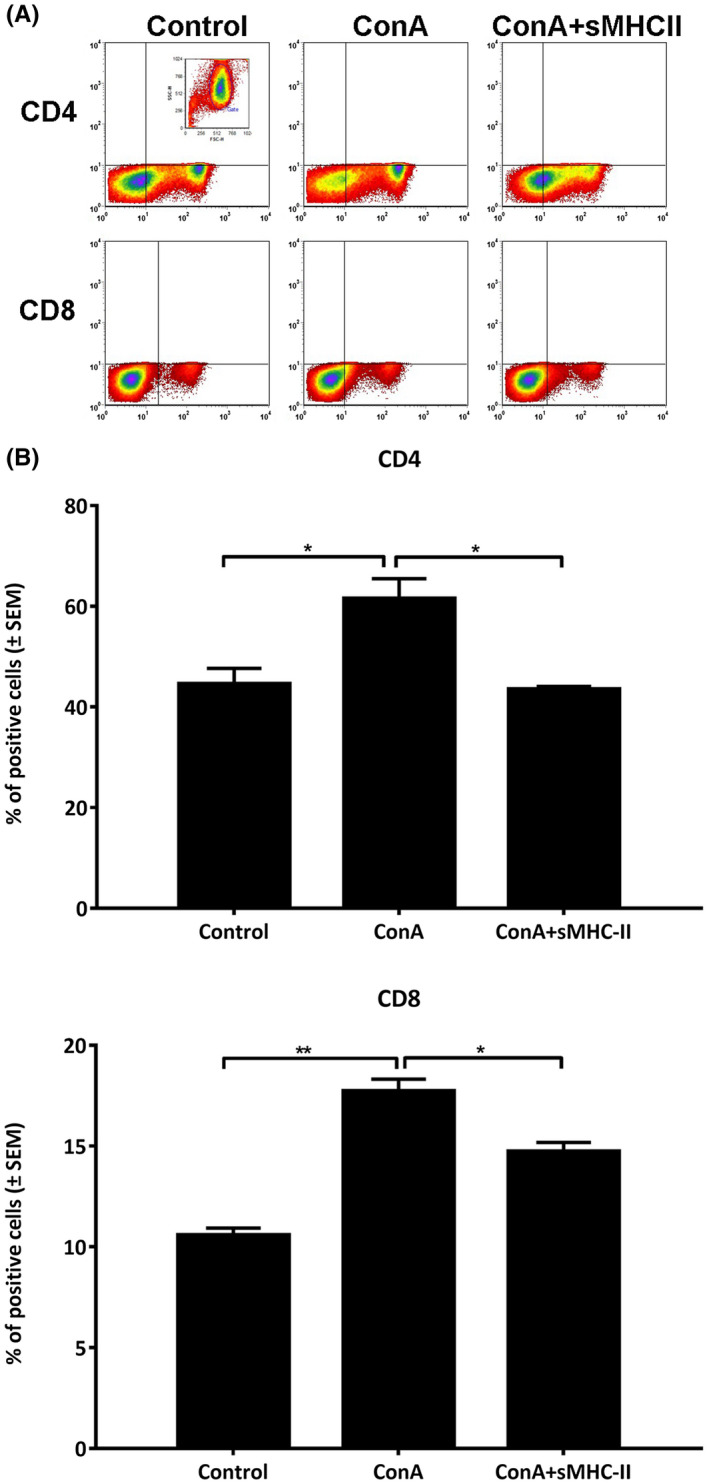
Effect of sMHCII administration on T‐cell marker expression of ConA‐treated animals. ConA‐treated animals received 0.3 μg/gr of body weight sMHCII and animals were sacrificed 3 h later. The percent of CD4^+^ and CD8^+^ cells in the spleen was evaluated by immunofluorescence followed by flow cytometry analysis (A). The results represent the mean of four mice in each case and are expressed as percent of positive cells ± SEM (B). *P < .02, **P < .002, ***P < .0005, ****P < .0001

Examining the effect of sMHCII administration on the CD4^+^ and CD8^+^ T‐cell populations, the animals that had received the 2‐week protocol of ConA treatment with or without sMHCII injection, were sacrificed 3 hours after the administration of sMHCII and spleen cells were processed to immunostaining, followed by flow cytometry analysis (Figure 5). Indeed, the results showed that the ConA treatment significantly increased the levels of CD4^+^ and CD8^+^ cells by 38 (*P* = .0246) and 67% (*P* = .0020), respectively, as compared to the untreated animals, while the administration of sMHCII proteins decreased by 29 (*P* = .0121) and 17% (*P* = .0270) the levels of CD4^+^ and CD8^+^ cells, respectively, as compared to the ConA‐treated animals (Figure 5).

### 3.4 Phenotypic evaluation of AIH establishment and rescuing

Following the 2‐week intraperitoneal ConA administration protocol, the animals were monitored as to their behavior for a period of 6 months. The overall assessment of murine behavior and well‐being was assessed by developing a scoring system to monitor the activity, mouse's coat condition (grooming), the response to stimulus, and the appearance of their genitals (Table 1). A 0‐3 score was established defining activity from normal (score 0) to slightly suppressed (score 1), suppressed (score 2), and no activity (score 3), grooming from clean fur and tail (score 0) to fur with yellow spots (score 1), yellow fur (score 2), and fur covered with urine fluids (score 3), response to stimulus from immediate response (score 0) to slow response to auditory stimulus (score 1), no response to auditory stimulus (score 2) and no response to auditory stimulus with mild response to touch (score 3) and the appearance of genitals from normal (score 0) to slightly swollen (score 1), atrophic and red (score 2), and bloody atrophic (score 3) (Table 1).

**TABLE 1:** Murine inducible AIH Score to assess the severity of disease in an experimental model of autoimmune hepatitis

Score	Description			
	Activity	Grooming	Response to stimulus	Genitals
0	Normal activity. Mouse is any of: eating, drinking, climbing, running, fighting	Mouse's both fur and tail look clean, absence of any odor	Mouse responds immediately to auditory stimulus or touch	Normal genitals
1	Slightly suppressed activity. Mouse is moving around the bottom of cage	Mouse's fur has quite yellow spots, in contrast with control mice	Slow or no response to auditory stimulus; strong response to touch (moves to escape)	Slightly swollen genitals with some indications of redness
2	Suppressed activity. Mouse is stationary with occasional investigative movements	Mouse's fur seems yellow, especially around the urinary system and the back, with unpleasant odor	No response to auditory stimulus; moderate response to touch (moves a few steps)	The genitals start looking atrophic and red
3	No activity. Mouse is stationary	Mouse's fur is covered in urine fluids, especially around the urinary system, presence of unpleasant odor	No response to auditory stimulus; mild response to touch (no locomotion)	Bloody, atrophic genitals

**FIGURE 6:**
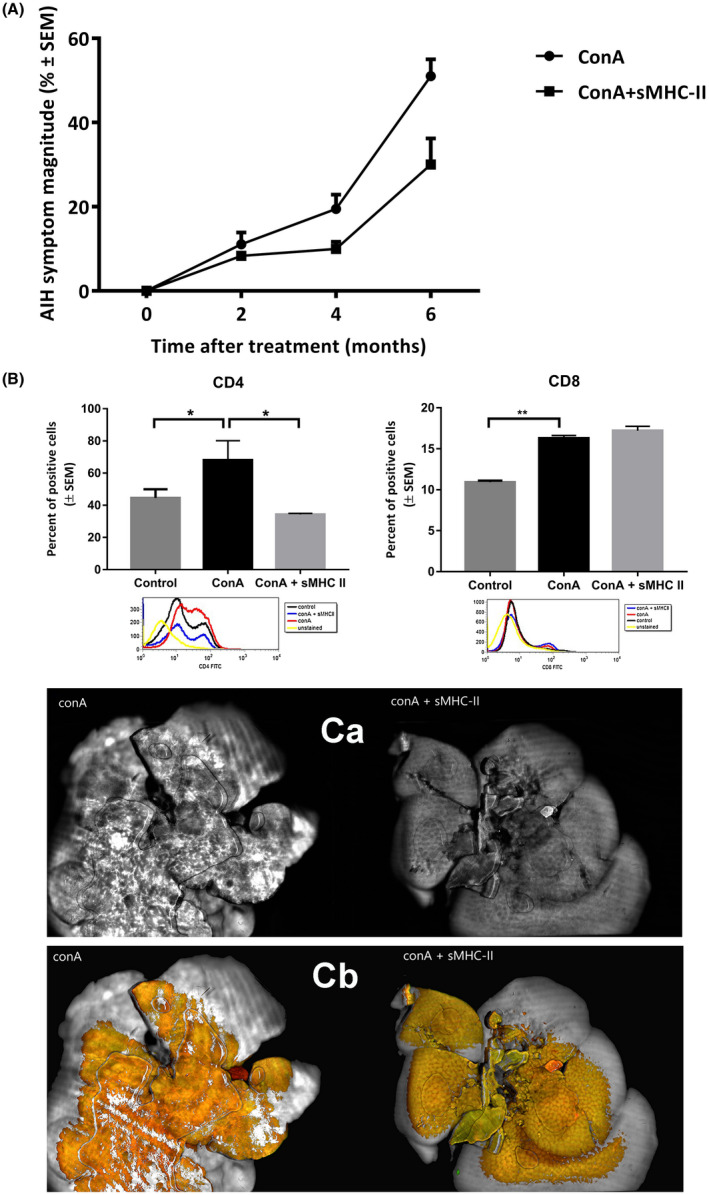
Effect of sMHCII administration on AIH‐symptom magnitude (A). Upon definition of the scoring rate regarding activity, mouse's coat condition (grooming), the response to stimulus and the appearance of their genitals (Table 1), mice were observed in a daily basis and behavior was recorded once a month for a period of 6 mo. The results represent the mean of five mice in each case and are expressed as the percent of AIH‐symptom magnitude with 100% corresponding to score 3 for all variables tested ± SEM. The effect of sMHCII administration on T‐cell marker expression of ConA‐treated animals after the period of 6 months was evaluated (B). ConA‐treated animals received 0.3 μg/gr of body weight sMHCII and animals were sacrificed 6 mo later. The percent of CD4^+^ and CD8^+^ cells in the spleen was evaluated by immunofluorescence followed by flow cytometry analysis (example histograms shown). The results represent the mean of five mice in each case and are expressed as percent of positive cells ± SEM. *P < .02, **P < .002. Livers from the ConA or ConA + sMHCII treated animals were submitted to scanning imaging (C). Livers were isolated, fixed and scanned using a white light beam at 10‐μm resolution (Ca) or four‐channel fluorescence at 10‐μm resolution (Cb)

Upon definition of the scoring rate, mice were observed in a daily basis and behavior was recorded once a month. Although serologically the effect of ConA administration and rescuing by sMHCII was apparent 3 hours after injection, the phenotypic establishment of AIH was a relatively slow procedure, manifesting the first symptoms 2 months after injection (Figure 6A). Six months after treatment initiation, the animals were showing severe AIH symptoms characterized by reduced activity, no response to auditory stimuli, lack of grooming and bloody atrophic genitals (Figure 6A). Importantly, the phenotypic symptoms of the mice having received the additional sMHCII treatment were significantly delayed in comparison to the ConA‐treated mice (Figure 6A). After the 6‐month period, ConA‐treated animals were still having increased levels of CD4^+^ and CD8^+^ cells (53%, *P* = .0355 and 53%, *P* = .0026, respectively) as compared to untreated mice, while the sMHCII treatment rescued only the CD4^+^ cell levels to control values (Figure 6B).

In order to evaluate the condition of the liver in the ConA‐ and the ConA‐sMHCII‐treated animals, tissues were isolated 6 months after the treatment, fixed and scanned using a white light beam (Figure 6Ca) or a four‐channel fluorescence (Figure 6Cb) biomolecular imager. Interestingly, the fibrotic/advanced necrotic appearance of the ConA‐treated livers was largely rescued by the administration of sMHCII proteins (Figure 6C), indicating that sMHCII molecules could be proposed as a therapeutic treatment for AIH even in humans.

## 4 DISCUSSION

Autoimmune hepatitis (AIH) is a severe autoimmune condition manifested by chronic liver inflammation leading to fibrosis and eventually necrosis. Among the several experimental animal models described in the literature, the present study concentrated on an inducible by ConA model of BALB/c mice, which has been shown to display most of the pathologic symptoms observed in humans^9^, ^10^ and attempted to rescue the AIH phenotype by sMHCII molecules, which as previously shown play an important role in maintaining tolerance to the organism.^21^ The results presented herein showed that indeed, serum‐isolated syngeneic sMHCII molecules could rapidly reverse all serologic and cellular markers tested to control levels, while alleviating in the long term the pathologic AIH phenotype.

The in vitro experimental model developed in this study consisted of normal spleen cell stimulation with a suboptimal immunogenic dose of ConA, where the presence of sMHCII proteins could significantly decrease cell proliferation as compared to ConA‐treated controls. Although very simple, these results showed that sMHCII molecules could suppress the ConA‐induced effect, and were therefore promising for their effective application in vivo.

Soluble MHCII were purified from normal syngeneic mouse serum and applied to the experimental model examined in this study. As mentioned in the introduction, sMHCII proteins are loaded with self/tolerogenic antigenic epitopes, maintaining thus tolerance by blocking autoreactive T‐cell activation.^21^, ^22^ Indeed, sMHCII molecules isolated from healthy/normal mice have revealed by mass spectroscopy analysis more than 600 self‐antigenic epitopes.^22^ Within that list of antigenic epitopes, one recognizes small nuclear ribonucleoprotein‐associated protein B, ribonucleoprotein A, isoform 2 of spectrin alpha chain, myosin‐9, myosin light chain, lamin‐A, anti‐mitochondrial autoantigens, stress‐70 protein, all of which are being recognized as autoantigens in a variety of AIH patients.^25^

The so far described in the literature ConA‐induced AIH model, applicable on BALB/c mice by intravenous injection has been characterized as an acute hepatitis model, whereas in humans AIH is generally characterized as a chronic disease.^27^ Therefore, in the present study, the ConA model was modified by changing the root of administration from intravenous to intraperitoneal. According to this modified model, the establishment of the cytokine inverse symptoms described in the classical ConA AIH model could be obtained by two weekly doses of ConA. Phenotypically, this model was much milder, since except from the serological symptoms, the animals were fully active during treatment. This slow development of the disease allowed application of the sMHCII treatment, which was given 3 hours after the second injection of ConA. The results were indeed very promising, because the administration of sMHCII proteins reversed the serological markers to almost control levels.

The immunological mechanisms of ConA‐induced AIH have been thoroughly studied, and it has been considered that the increase of blood levels of IL‐2, IL‐4, and IFN‐γ implicates the involvement of CD4^+^ cells in the liver injury, while CD8^+^ cells were also considered to contribute to injury by target cell lysis.^9^ In the modified ConA model defined herein, cytokines IL‐2, IL‐4 as well as CD4^+^ and CD8^+^ cells were found to be increased and interestingly, their levels were rescued by the administration of sMHCII. An additional issue that was addressed was the role of IL‐10 in the process of injury. In the present study, the levels of IL‐10 significantly increased after the second injection of ConA and decreased to control levels upon administration of sMHCII. IL‐10 is an anti‐inflammatory cytokine, which at early onset of the injury could suppress protective immunity and therefore aggravate injury.

Following the ConA‐modified AIH model, the first phenotypic symptoms of the disease were apparent 2 months after the treatment. In order to evaluate disease aggravation, a scoring system ranging from 0 to 12 was developed based on the activity of the animals, their willingness to grooming, the response to stimulus and the appearance of their genitals. The animals were followed up to 6 months reaching a score of 8, and their condition was worsening to terminal stage in most cases by 8 months. Most importantly, liver examination in the ConA‐treated animals showed a cirrhotic/necrotic appearance, whereas the treatment with sMHCII largely rescued this appearance, and livers were showing a healthy morphology.

In conclusion, the results presented in the present report promote once more the ability of sMHCII molecules to rescue the pathologic symptoms of AIH. In addition to the beneficial effect of sMHCII molecules in alleviating SLE symptoms,^23^ these serum extracts could be proved to be a therapeutic solution for autoimmune diseases. If, as in the case of SLE,^23^ allogeneic sMHCII could be definitely shown to be as effective as the syngeneic ones, these molecules could be of great value in therapy.

## CONFLICT OF INTEREST

None.

## AUTHOR CONTRIBUTIONS

KB supervised and validated the laboratory work performed by MGD and ES, and wrote the original draft of the paper in collaboration with MGD. IA had the overall responsibility and supervision of the work and wrote the final version of the manuscript.
